# Tetraploidy-associated centrosome overduplication in mouse early embryos

**DOI:** 10.1080/19420889.2018.1526605

**Published:** 2018-10-02

**Authors:** Kan Yaguchi, Takahiro Yamamoto, Ryo Matsui, Masaya Shimada, Atsuko Shibanuma, Keiko Kamimura, Toshiaki Koda, Ryota Uehara

**Affiliations:** a Graduate School of Life Science, Hokkaido University, Sapporo, Japan; b Creative Research Institution, Hokkaido University, Sapporo, Japan; c Faculty of Advanced Life Science, Hokkaido University, Sapporo, Japan

**Keywords:** Tetraploid, centrosome, mouse embryo

## Abstract

Recently, we observed that tetraploidization of certain types of human cancer cells resulted in upregulation of centrosome duplication cycles and chronic generation of the extra centrosome. Here, we investigated whether tetraploidy-linked upregulation of centrosome duplication also occurs in non-cancer cells using tetraploidized parthenogenetic mouse embryos. Cytokinesis blockage at early embryonic stage before de novo centriole biogenesis provided the unique opportunity in which tetraploidization can be induced without transient doubling of centrosome number. The extra numbers of the centrioles and the centrosomes were observed more frequently in tetraploidized embryos during the blastocyst stage than in their diploid counterparts, demonstrating the generality of the newly found tetraploidy-driven centrosome overduplication in mammalian non-cancer systems.

Mammalian somatic cells are usually diploid, consisting of two pairs of genome, and generally intolerant to alterations in their DNA content. Tetraploidization (whole genome duplication) because of cell division failure often impairs genome integrity in mammalian somatic cells and potentially leads to tumorigenesis or developmental deficiencies [1–8]. The mechanism via which tetraploidization affects genome integrity and other basic biological processes remains poorly understood. A recent study showed that doubling of chromosome number upon tetraploidization increases the chances of chromosome aberrations and cellular tolerance to aneuploidy, potentially promoting expansion of cell population with chromosomal abnormalities [9]. Another possible cause of tetraploidy-linked cellular defects is doubling of centrosome number, which accompanies cell division failure and adversely affects proper mitotic regulation through multipolar spindle formation [10]. The extra centrosome gradually disappears from the polyploidized cell population possibly because of its disadvantageous effect on cell viability, but is possibly continuously harbored in certain populations because of adaptative centrosome clustering or its advantageous effect on cellular invasiveness [11–13]. Consistent with these ideas, the extra centrosome is observed in majority of tumor cells and cancer cell lines with chromosomal instability [14]. Interestingly, we recently observed that tetraploidized cell lines derived from near haploid or diploid cancer cells showed accelerated centrosome duplication compared to their haploid or diploid counterparts, which led to chronic extra centrosome generation [15]. Based on this observation we proposed that this tetraploidy-driven centrosome overduplication contributes to genome instability in the tetraploid state. However, it remains to be determined whether the tetraploidy-driven centrosome overduplication potentially takes place in non-cancer systems such as early embryos, especially considering the fact that the tetraploidy-linked extra centrosome was not observed in tetraploidized cells derived from a normal immortalized epithelial cell line hTERT-RPE1 cells [15]. It is also unclear whether the extra centrosome arises in tetraploid cells without the initial doubling of centrosome number after tetraploidization solely from the tetraploidy-driven upregulation of centrosome duplication. A possible experimental approach to address this latter issue would be to induce tetraploidization without doubling centrosomes.

During mammalian gametogenesis, primary oocytes lose their centrioles and retain them only during the late pre-implantation stage (e.g. E3.5 – E4.5 in mouse embryos) post-fertilization through de novo centriole biogenesis [16–18]. Therefore, the induction of cell division failure during this pre-centriole stage produces tetraploid embryos without supernumerary centrosome formation. Here, we used this unique property of centriole biogenesis in mouse early embryos to investigate the impact of tetraploidy on centrosome number control in non-cancer cells without the contributions of the extra centrosome brought in upon induction of tetraploidization.

To investigate the impact of tetraploidy on centrosome number control, we generated mouse tetraploid embryos from diploid parthenogenetic embryos [19]. Inhibition of the second cleavage in diploid embryos during E1.5 stage by cytochalasin B treatment resulted in the formation of embryos with two binucleated interphase cells at E2.0 stage (38 out of 38 developing embryos from two independent experiments; Figure 1(a,b)). The majority of embryos at this stage were devoid of Cep135-positive structure corresponding to the centriole (6 out of 7 diploid embryos, and 7 out of 7 tetraploid embryos from two independent experiments), indicating that de novo centriole biogenesis had not occurred at the time of tetraploidization [17,18]. Estimation of chromosome number by kinetochore counting in embryonic cells, which were mitotically arrested using an Eg5 inhibitor, S-trityl-L-cysteine (STLC), for 24 h from E3.5 to visualize individual sister chromatids [20], showed that cells in the tetraploidized embryos maintained their tetraploid DNA content until this embryonic stage (Figure 1(c,d)). Finally, we performed immunostaining of Cep135 and γ-tubulin, which mark the centriole and the centrosome, respectively, in diploid and tetraploid parthenogenetic embryos fixed at E4.5 (Figure 1(e,f)). The frequency of cells that possessed the supernumerary centrioles and centrosomes was significantly higher in the tetraploidized embryos than in their diploid counterparts. This result clearly demonstrates that tetraploidy-driven centrosome overduplication observed in human cancer cell lines also occurs in non-cancer mouse embryonic cells, suggesting the generality of the newly identified pathway of centriole deregulation. Tetraploidization in early embryos usually leads to severe developmental deficiencies in mammalian species [8]; however, the identity of the cellular processes that are affected by tetraploidization remain unclear. Our observation indicates that tetraploidization of early mammalian embryonic cells, even when it occurs prior to centriole possession, damages the subsequent centrosome number control and thereby potentially perturbs the genetic stability of their progenies.

**Figure 1.**
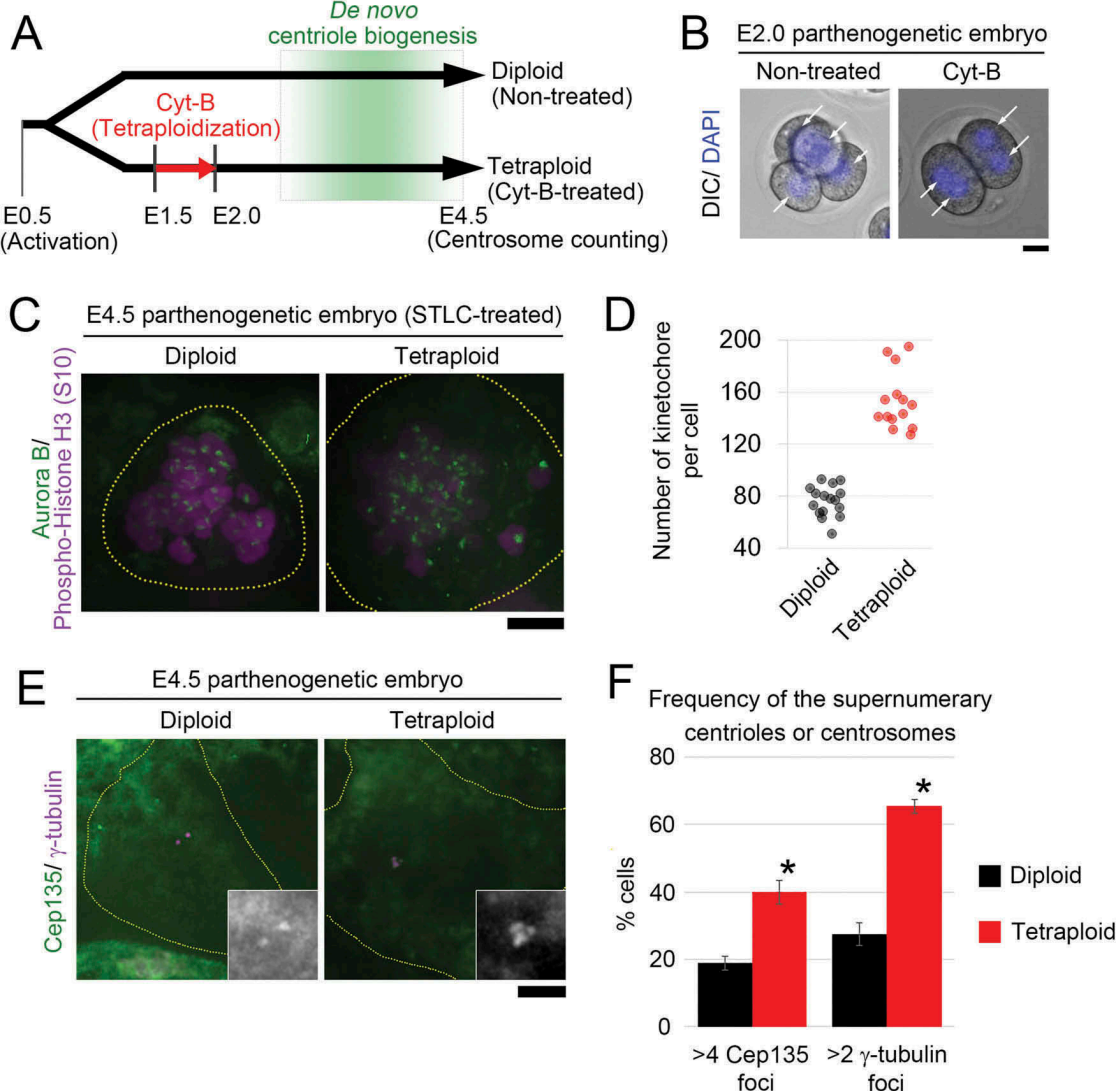
Frequent generation of the supernumerary centrosome in tetraploidized embryos. (a) A schematic of the experimental procedure. (b) Microscopy of E2.0 embryos treated with or without cytochalasin B for 12 h. DNA was visualized by 4ʹ,6-diamidino-2-phenylindole (DAPI). Arrows indicate nuclei. Representative images from two independent experiments are shown. Scale bar, 20 µm. (c) Immunofluorescence of phosphorylated histone H3 (S10) and aurora B, which mark mitotic chromosomes and kinetochores, respectively, in cells in diploid or tetraploidized parthenogenetic embryos mitotically arrested by STLC treatment. Broken lines mark cell boundaries. Scale bar, 5 µm. (d) Estimation of kinetochore numbers in (C). Sixteen cells in eight diploid embryos and 14 cells in 10 tetraploid embryos from two independent experiments were analyzed. Only cells with clear kinetochore staining were used for quantification; the remaining unquantified cells had cell size and chromosome mass similar to those of quantified cells. (e) Immunofluorescence of Cep135 and γ-tubulin, which mark the centriole and centrosome, respectively, in interphase cells in E4.5 diploid or tetraploid parthenogenetic embryos. Scale bar, 5 µm. Broken lines mark cell boundaries. Insets show 2× enlarged images of centrioles. (f) Frequency of supernumerary centrioles and centrosomes in (E). Mean ± SE of three independent experiments (*p < 0.05, two tailed paired *t*-test). One hundred ninety cells in 17 diploid embryos, and 149 cells in 25 tetraploid embryos were analyzed.

## Materials and methods

### Parthenogenesis, and embryo culture

Mouse diploid parthenogenic embryos were generated as previously described with slight modifications [21,22]. Eight–12-week-old female B6D2F1 (C57BL/6 × DBA/2) (Japan SLC, Inc.) were injected with 5 IU pregnant mare serum gonadotropin (PMSG, ASKA Animal Health) followed by injection with 5 IU human chorionic gonadotropin (hCG, ASKA Pharmaceutical) 46–48 h later, and matured oocytes were obtained from oviducts 16 h later. Oocytes were treated with 0.1% hyaluronidase (Sigma-Aldrich) in M2 medium (Sigma-Aldrich) for 1 min to remove cumulus cells, washed with M2 medium and M16 medium (Sigma-Aldrich) three times each, and incubated in M16 medium supplemented with 2 mM EGTA (EGTA-M16) for 20 min. Diploid parthenogenetic embryos were produced by treating the oocytes with 2 mM SrCl_2_ in EGTA-M16 in the presence of 5 µg/mL cytochalasin B (Wako) for 2.5 h, and then incubating them in KSOM medium (MTI-GlobalStem) in the presence of the same concentration of cytochalasin B for 3.5 h. Activated diploid parthenogenetic embryos were then washed with KSOM three times and cultured in the same medium at 37°C with 5% CO_2_. In case diploid embryos were converted to tetraploid after parthenogenesis, E1.5 embryos were treated with 5 µg/mL cytochalasin B for 12 h to block the second cleavage, and washed three times with KSOM.

### Ethics statement

The maintenance and handling of mice for all embryo experiments were performed in the animal facility of the Platform for Research on Biofunctional Molecules of Hokkaido University under the guidelines and with the permission of the committee on animal experiments of Hokkaido University (permission number 16–0038).

### Antibodies

Antibodies were purchased from suppliers, and used at dilutions, as follows: mouse monoclonal anti-γ-tubulin (GTU88, Sigma-Aldrich; 1:100 or 1:50); rabbit polyclonal anti-Cep135 (ab75005, Abcam; 1:50); mouse monoclonal anti-aurora B (611082, BD Biosciences; 1:50); rabbit polyclonal anti-phospho-histone H3(S10) (A301-844A, Bethyl Laboratories; 1:50); and fluorescence-conjugated secondaries (Jackson ImmunoResearch Laboratories; 1:100 or 1:50).

### Immunofluorescence staining

For immunofluorescence staining embryos were fixed with 100% methanol at −20°C for 10 min. Fixed samples were treated with BSA blocking buffer (150 mM NaCl; 10 mM Tris-HCl, pH 7.5; 5% BSA; and 0.1% Tween 20) for 30 min at 25°C, incubated with primary antibodies for 36–48 h at 4°C, and incubated with secondary antibodies for 36–48 h at 4°C. Following each treatment, embryos were washed 2–3 times with PBS. Stained mouse embryos were embedded in 0.5% PrimeGel Agarose LMT (Takara Bio) dissolved in DPBS.

### Microscopy

Cells were observed under a TE2000 microscope (Nikon) equipped with a × 100 1.4 numerical aperture (NA) Plan-Apochromatic, a × 60 1.4 NA Plan-Apochromatic, or a × 40 1.3 NA Plan Fluor oil immersion objective lens (Nikon), a CSU-X1 confocal unit (Yokogawa), and an iXon3 electron multiplier-charge coupled device (EMCCD) camera (Andor) or an ORCA-ER CCD camera (Hamamatsu Photonics). Image acquisition was controlled by µManager software (Open Imaging).
